# Long Non-coding RNA AGAP2-AS1 Silencing Inhibits PDLIM5 Expression Impeding Prostate Cancer Progression *via* Up-Regulation of MicroRNA-195-5p

**DOI:** 10.3389/fgene.2020.01030

**Published:** 2020-09-25

**Authors:** Pingbo Xie, Mingsheng Liu, Fen Chen, Shaomei Wu, Tao Shao, Wei Wang, Chenxiang Xu, Hongqing Zhou

**Affiliations:** The Second Ward of Urology, Qujing Affiliated Hospital of Kunming Medical University, Qujing, China

**Keywords:** long non-coding RNA AGAP2-AS1, micorRNA-195-5p, prostate cancer, PDZ and LIM domain 5, competing endogenous RNA

## Abstract

Prostate cancer remains a significant cause of cancer-related deaths in male population. More recently, accumulating evidence continues to implicate long non-coding RNAs (lncRNAs), microRNAs (miRNAs), and mRNAs in various types of cancers, including prostate cancer. The current study aimed to elucidate the role of lncRNA AGAP2-AS1/miR-195-5p/PDZ and LIM domain 5 (PDLIM5) in prostate cancer progression. Initially, microarray expression profiles were applied to screen differentially expressed lncRNAs/miRNAs/genes associated with prostate cancer. Dual-luciferase reporter and RNA pull-down/RIP assays were subsequently performed to explore the interactions among lncRNA AGAP2-AS1, miR-195-5p, and PDLIM5, after which their expression was detected in cancer tissues and cells. Next, gain- and loss-of-function approaches were employed to elucidate the mechanism of lncRNA AGAP2-AS1/miR-195-5p/PDLIM5 in the processes of cell proliferation, migration and invasion as well as tumor growth. LncRNA AGAP2-AS1 was found to be highly expressed in prostate cancer. Silencing of lncRNA AGAP2-AS1 contributed to the suppression of proliferation, migration and invasion of cancer cells *in vitro*. Besides, lncRNA AGAP2-AS1 could bind to miR-195-5p which targeted PDLIM5 and subsequently downregulated its expression, ultimately impeding the progression of prostate cancer. Additionally, lncRNA AGAP2-AS1 inhibition led to an up-regulated expression of miR-195-5p and down-regulated PDLIM5 expression, resulting in delayed tumor growth *in vivo*. Taken together, the key findings of our study demonstrated that lncRNA AGAP2-AS1 silencing exerted suppressive effects on the development of prostate cancer via the miR-195-5p-dependent downregulation of PDLIM5. Our findings highlighted the potential of lncRNA AGAP2-AS1 as a promising novel molecular target for prostate cancer therapy.

## Introduction

As one of the most common cancers afflicting the male population, prostate cancer was previously reported to account for 1.2 million new cases diagnosed in 2018 (Bray et al., 2018). The greater majority of prostate cancers remain dormant or inactive for a considerable length of time, but metastatic progression is likely to rapidly lead to poor prognosis and death (Birnbaum et al., 2019). Prostate cancer treatment has witnessed substantial improvements over the past decade with novel therapeutics, improved functional imaging, and an improved application of existing therapies all reported to improve patient outcome (Teo et al., 2019). In spite of the advanced application of known risk factors, genetic testing represents a comparatively underutilized factor in prostate cancer (Fantus and Helfand, 2019). Thus, it is critical to highlight the need for further elucidation of the finer molecular mechanisms associated with prostate cancer, which may help to identify novel diagnostic and therapeutic targets.

Long non-coding RNAs (lncRNAs) represent a type of transcripts longer than 200 nucleotides, and their aberrant expression is associated with different cancer types due to the crucial modulatory role in tumor formation as well as cancer development (Huarte, 2015). LncRNA AGAP2 antisense RNA 1 (AGAP2-AS1) is a lncRNA, 1567 nt in length, transcribed from another gene which is located at 12q14.1, and highly-expressed lncRNA AGAP2-AS1 has been implicated in many human cancers (Dong et al., 2018). Additionally, lncRNA AGAP2-AS1 has been reported to be differentially expressed in patients with non-small cell lung cancer (NSCLC), and its up-regulation has been suggested to promote the development and progression of NSCLC (Li et al., 2016; Qi et al., 2017). LncRNAs antagonize the post-transcriptional regulation of microRNAs (miRNAs/miRs) in gene expression during disease development by means of acting as competing endogenous RNAs (ceRNAs), (Tay et al., 2014).

miRNAs represent endogenous non-protein-coding small noncoding RNAs, 18–25 nucleotides in length, that have been implicated in a wide array of tumor cell physiological processes including cell proliferation, invasion, apoptosis, and self-renewal by interacting with their specific target genes (Wang et al., 2017; Li et al., 2018). Furthermore, accumulating evidence has suggested that miRNAs play a crucial role in regulating tumor development (Ling et al., 2013; Rupaimoole and Slack, 2017). For instance, miR-195-5p has been demonstrated to be an important tumor suppressor in cases of NSCLC and prostate cancer (Cai et al., 2018; Zheng et al., 2019). The Targetscan website revealed the presence of binding sites of miR-195-5p in the 3′untranslated region (3′UTR) of PDZ and LIM domain 5 (PDLIM5) mRNA. PDLIM5, also known as enigma homolog, represents a type of scaffold protein which contains a PDZ domain at the N-terminal end and three C-terminal LIM domains (Ito et al., 2015; Cheng et al., 2016). The PDLIM5 gene has been previously demonstrated to exert an influence on major depressive disorder, schizophrenia, alcohol dependence, bipolar disorder and cancer (Owusu et al., 2017; Wei et al., 2018). Furthermore, PDLIM5 has been reported to be abnormally enriched in prostate cancer tissues, with its knockdown shown to trigger suppression of proliferative, migratory and invasive abilities of prostate cancer cells (Liu et al., 2017). In line with the exploration of the aforementioned literature, we hypothesized that lncRNA AGAP2-AS1 plays a critical role in prostate cancer development with miR-195-5p and PDLIM5 playing a contributory role. Hence, the central objective of the current study was to investigate the relationships among lncRNA AGAP2-AS1, miR-195-5p, and PDLIM5, and their regulatory mechanism influencing cell proliferation, migration and invasion of prostate cancer cells.

## Materials and Methods

### Ethics Statement

The study was performed with the approval of the Ethics Committee of Qujing Affiliated Hospital of Kunming Medical University. The study was performed in strict accordance with the *Declaration of Helsinki*. All patients or their family were informed of the research purposes and provided their written informed consent prior to enrollment. Animal experiments were conducted with the approval of the Animal Ethics Committee of Qujing Affiliated Hospital of Kunming Medical University. Extensive efforts were made to ensure minimal suffering of the included animals.

### Bioinformatics Analysis

Prostate cancer-related gene expression profile GSE34933 and annotated probe files were initially downloaded from the Gene Expression Omnibus (GEO)^1^ and processed using the Affymetrix Human Genome U133 Plus 2.0 Arrays, followed by background correction and normalization via the Affy package of the R software (Fujita et al., 2006). Differentially expressed lncRNAs were then screened from non-specific filtered the expression profile data using the linear model-Empirical Bayes method in the “limma” package combined with the traditional *t*-test (Smyth, 2004). miRNAs screened out from the redundant arrays of inexpensive disks (RAID) architectures were intersected with the lowly-expressed miRNAs in prostate cancer from GSE34933 microarray data. Finally, the target genes of the candidate miRNA predicted from the miRDB, TargetScan7, starBase and mirwalk databases were intersected with over-expressed genes in prostate cancer from the GSE103512 microarray data.

### Tissue Sample Collection

Prostate cancer tissues were excised from 50 male prostate cancer patients with additional tissue specimens collected from 20 individuals diagnosed with benign prostatic hyperplasia (BPH) from Qujing Affiliated Hospital of Kunming Medical University. During prostatectomy, a prostate cancer tissue block and a BPH tissue block (about 1 cm^2^) of the patient were collected in an RNA locker (GT21233, Shanghai Xinyu Biological Technology Co., Ltd., Shanghai, China), and subsequently stored in a −80°C refrigerator after incubation at 4°C overnight. The 50 males with prostate cancer were aged between 50 and 88 years with a median age of 68 years. Based on the imaging results before radiotherapy, these patients were classified in accordance with the 2014 International Society of Urological Pathology (ISUP) Gleason grading system (Epstein et al., 2017). The population consisted of 5 cases of Gleason grade 1, 14 cases of Gleason grade 2, 9 cases of Gleason grade 3, 14 cases of Gleason grade 4 and 8 cases of Gleason grade 5. All cases were diagnosed with primary prostate cancer based on pathological examination. There was no distant metastasis in all patients with prostate cancer. None of the patients in the study had received any radiotherapy or chemotherapy prior to surgery.

The 20 male BPH patients were aged 53–86 years, with a median age of 69 years. These patients had not undergone urethral catheterization or digital rectal examination prior to surgery. They had not received any androgen-related drug therapy. No prostate tumor or no prostate proximal cancer cell hyperplasia was observed in the enrolled BPH patients.

### Immunohistochemistry

The positive expression of PDLIM5 protein in tissues was determined using the streptavidin-perosidase (SP) immunohistochemical staining method. More specifically, paraffin-embedded sections were deparaffinized without antigen retrieval, then directly immersed in 3% H_2_O_2_ to block the endogenous enzyme activity for 10 min and washed 3 times with phosphate buffered saline (PBS). The sections were subsequently blocked using 10% normal goat serum for 10 min followed by incubation with primary rabbit anti-human PDLIM5 antibody (1 : 500, ab83060, Abcam Inc., Cambridge, United Kingdom) for 1 h, followed by additional 10-min incubation with biotin-labeled secondary antibody goat anti-rabbit Immunoglobulin G (IgG) (1:1000, ab6720, Abcam Inc., Cambridge, United Kingdom). Finally, the sections were added with SP solution for 10 min, and then developed with diaminobenzidine (DAB). After counterstaining with hematoxylin, the sections were mounted with neutral balsam and observed under a microscope.

### Reverse Transcription Quantitative Polymerase Chain Reaction (RT-qPCR)

Total RNA was extracted from tissues and cells using TRIzol reagents (Invitrogen Inc., Carlsbad, CA, United States). The RNAs (miRNA or mRNA) were separately reversely transcribed into complementary DNA (cDNA) via the stem-loop method [All-in-One^TM^ miRNA First-Strand cDNA Synthesis Kit (GeneCopoeia Inc., Rockville, MD, United States), and an oligo (dT) primer (PrimeScript^TM^ 1st Strand cDNA Synthesis Kit, Takara Bio Inc., Dalian, China]. SYBRgreen PCR assay was performed in accordance with the following method: pre-denaturation at 95°C for 3 min, a total of 40 cycles of denaturation at 95°C for 15 s, annealing at 60°C for 30 s, and extension at 72°C for 20 s, and a final extension at 72°C for 5 min. All utilized primers (Table 1) were synthesized by Beijing Genomics Institute (BGI) (Beijing, China). The reliability of the PCR results was evaluated based on a solubility curve and calculated using the relative quantitative method. The relative expression of each target gene was expressed by 2^–ΔΔCt^ method (Schefe et al., 2006). The experiment was performed in triplicate.

**TABLE 1:** Primer sequences for reverse transcription quantitative polymerase chain reaction.

**Gene**	**Primer sequence**
PDLIM5	F: 5′-CCGGTTCCTGTTCAAAAGGG-3′
	R: 5′-GCCGTGGTGCCTTATTGTAG-3′
miR-195-5p	F: 5′-UAGCAGCACAGAAAUAUUGGC-3′
	R: 5′-GCCAATATTTCTGTGCTGCTA-3′
LncRNA AGAP2-AS1	F: 5′-TACCTTGACCTTGCTGCTCTC-3′
	R: 5′-TGTCCCTTAATGACCCCATCC-3′
MMP2	F: 5′-TTGATGGCATCGCTCAGATC-3′
	R: 5′-TTGTCACGTGGCGTCACAGT-3′
cyclin	F: 5′-TCCATGGCAGGGAACTIT-3′
	R: 5′-AGATTGGOCTGTAGCTAGA-3′
U6	F: 5′-CTCGCTTCGGCAGCACA-3′
	R: 5′-AACGCTTCACGAATTTGCGT-3′
GAPDH	F: 5′-GCCAAGGTCATCCATGACAACT-3′
	R: 5′-GAGGGGCCATCCACAGTCTT-3′

*F, forward; R, reverse; PDLIM5, PDZ and LIM domain 5; miR-195-5p, microRNA-195-5p; LncRNA AGAP2-AS1, long non-coding RNA AGAP2 antisense RNA 1; MMP2, matrix metalloproteinase 2; GAPDH, glyceraldehyde-3-phosphate dehydrogenase.*

### Cell Culture

Prostate cancer cell lines VCaP, 22Rv1, CRL-1740, CRL-2422, PC3M and normal prostate stromal cell WPMY-1 were purchased from the Cell Bank of Chinese Academy of Sciences (Shanghai, China). Following resuscitation, the cells were cultured with Roswell Park Memorial Institute (RPMI) 1640 medium (Gibco Inc., Carlsbad, CA, United States) containing 10% fetal bovine serum (FBS; Gibco) in an incubator (Thermo Fisher Scientific, MA, United States) in a saturated humidity atmosphere with 5% CO_2_ at 37°C. After cell confluence had reached 90%, the cells were detached with 0.25% trypsin and passaged at a ratio of 1:3.

### Cell Transfection

The cells in each group were plated into 6-well plates at a density of 10^5^ cells/well, and transfected with lncRNA AGAP2-AS1 overexpression (pCDNA-AGAP2-AS1), PDLIM5 overexpression (pCDNA-PDLIM5), miR-195-5p mimic, miR-195-5p inhibitor, small interfering RNA (siRNA) against PDLIM5 (si-PDLIM5), or si-AGAP2-AS1 in accordance with the instructions of the Lipofectamine 2000 reagent kit (Invitrogen Inc., Carlsbad, CA, United States) after cell confluence had reached 80%. The cells were then cultured in complete medium for 48 h and collected. The overexpression constructs were designed and constructed by Shanghai Gene Pharmaceutical Co., Ltd. (Shanghai, China). The siRNAs against AGAP2-AS1 (si-AGAP2-AS1) and PDLIM5 (si-PDLIM5), as well as their controls, were designed and synthesized by Thermo Fisher Scientific (Waltham, MA, United States). Negative control (NC)-mimic, NC-inhibitor, miR-195a-5p mimic, and miR-195a-5p inhibitor were purchased from Biomics Biotechnologies Co., Ltd. (Jiangsu, China).

### Western Blot Analysis

Protein was extracted from cells that were lysed using a radioimmunoprecipitation assay (RIPA) lysis buffer (P0013B, Beyotime Institute of Biotechnology, Shanghai, China) by means of high speed centrifugation. The concentration of the extracted protein was determined by bicinchoninic acid (BCA) assay (Pierce-Thermo Fisher Scientific, Waltham, MA, United States). The protein was then separated via 4 and 10% concentrated gel electrophoresis and transferred onto a membrane which was subsequently blocked using 5% bovine serum albumin (BSA). The membrane was incubated overnight at 4°C with rabbit anti-human antibodies (Abcam Inc., Cambridge, United Kingdom) to PDLIM5 (1:500, ab83060), glyceraldehyde-3-phosphate dehydrogenase (GAPDH) (1:1000, ab8245), matrix metalloproteinase 2 (MMP2) (1: 1000, Ab92536), or cyclin (1:1000, ab32053). The membrane was then washed, and incubated with horseradish peroxidase (HRP)-labeled polyclonal goat anti-rabbit IgG antibody (1:3000, ab6721, Abcam Inc., Cambridge, United Kingdom) for 2 h at room temperature. The membrane was subsequently developed with enhanced chemiluminescence (ECL) reagents (Invitrogen Inc., Carlsbad, CA, United States), imaged by Bio-Rad imaging system, and analyzed by the Image Lab software.

### Dual-Luciferase Reporter Assay

The lncRNA AGAP2-AS1 promoter sequence and gene full sequence were obtained from Gene database^3^. The pMIR-PDLIM5 (Miaolingbio. Inc., Hubei, China) [wild type [(wt): UUGCUGCUG/mutant (mut) with recognizing sites mutated: GGCUGGUGC] plasmids (400 ng) or pMIR-AGAP2-AS1 (wt: UGCUGCU/mut: GUUGUUC) plasmids (400 ng) were co-transfected with miR-195-5p mimic or NC into cells. Next, 20 μL cell lysate was mixed with 100 μL LARII solution in a 1.5 mL centrifuge tube based on the instructions of the luciferase reporter assay kit (Promega Corporation, Madison, WI, United States), with the luciferase activity detected using a luminometer (Promega Corporation, Madison, WI, United States) to detect the fluorescence value of OD460. The experiments were repeated three times in an independent manner.

### Cell Proliferation Assessment

Cells in the exponential phase were collected, and seeded in 96-well plates at a density of 3 × 10^4^ cells/mL (200 μL/well; marginal wells filled with sterile PBS; 6 replicate wells/group), and cultured in an incubator for 12 h. At 1, 2, 3, and 4 days of culture, each well was added with 20 μL cell counting kit-8 (CCK-8) solution (HyClone Company, Logan, UT, United States), and incubated at 37°C for a period of 2 h. The optical density (OD) value of each well at a wavelength of 450 nm was measured using a microplate reader.

### Scratch Test

The cells at the logarithmic phase were seeded in 6-well plates at a density of 10^6^ cells/well, with 15 uniformly distributed horizontal lines drawn on the back region using marker pens. After completely covering the surface of the 6-well plates, the cells were cultured in 1% FBS medium and starved for 12 h. A 200 μL pipette tip was applied to create a perpendicular scratch to the horizontal lines on the 6-well plates with a ruler. The scratched cells were washed 3 times using 2 mL PBS. Photographs were taken at 0 and 24 h. The width of the scratches across the horizontal lines was measured and the average width of the scratches was calculated. Cell mobility (%) = (1 − scratch width/initial scratch width) × 100%.

### Transwell Assay

Matrigel (Becton, Dickinson and Company, Franklin Lakes, NJ, United States) mixed with serum-free medium at a ratio of 1 : 1 was added to a Transwell chamber (Corning Incorporated, NY, United States) with a volume of 50 μL/well, and polymerized in a 37°C incubator. The cells in the exponential phase were treated with culture solution containing 1% FBS for 24 h. After detachment, 50 μL of cell suspension (1 × 10^6^ cells/mL) which had been previously mixed with 50 μL culture solution containing 2% FBS was added to the apical Transwell chamber, and 600 μL culture solution containing 10% FBS was added to the basolateral chamber and incubated in an incubator at 37°C with 5% CO_2_, saturated humidity and sufficient oxygen for 24 h. The cells were subsequently fixed in 4% paraformaldehyde and stained with crystal violet. Five visual fields were captured using the five-point sampling method with an inverted high-power microscope (400×) for counting.

### RNA-Binding Protein Immunoprecipitation (RIP) Assay

The binding of lncRNA AGAP2-AS1 to Argonaute 2 (Ago2) protein was detected using a RIP kit (Millipore Corp., Boston, MA, United States). In short, VCaP cells were washed with pre-cooled PBS, lysed by an equal volume of lysis buffer [25 mmol/L Tris-HCl buffer (pH 7.5), 150 mmol/L KCl, 2 mmol/L ethylene diamine tetraacetic acid (EDTA), 0.5% nonidet P 40 (NP40), 1 mmol/L sodium fluoride (NaF), 1 mmol/L dithiothreitol (DTT), 100 U/mL ribonuclease inhibitor (RNasin), EDTA-free protease inhibitor] in an ice bath for 5 min, and centrifuged at 14000 rpm and 4°C for 10 min. A total of 50 μL magnetic beads were resuspended in 100 μL RIP washing buffer and swirled. An eppendorf tube was placed on a magnetic stand and rotated left and right by 15° to make the magnetic beads align in a straight line. After the supernatant had been discarded, magnetic beads were resuspended with 100 μL RIP washing buffer, and added with 5 μg antibodies. The magnetic bead-antibody complex was washed and resuspended in 900 μL RIP washing buffer before incubation with cell lysate. A portion of the cell lysate was taken as an input, while another portion (100 μL) was co-precipitated via incubation with magnetic bead-antibody complex overnight at 4°C. The co-precipitated magnetic bead-protein complex and input were separately detached with proteinase K to extract RNA for subsequent RT-qPCR detection of lncRNA AGAP2-AS1. The antibody used for RIP was rabbit anti-human Ago2 (1:500, ab186733, Abcam Inc., Cambridge, United Kingdom), and rabbit anti-human IgG (1:500, ab109489, Abcam Inc., Cambridge, United Kingdom) was used as the NC.

### RNA Pull-Down Assay

As per the instructions of the magnetic RNA-protein pull-down kit (Pierce Biotechnology Inc., Rockford, IL, United States), an eppendorf tube added with 1 μg biotin-labeled miR-195a-5p (synthetized by Pierce Biotechnology Inc., Rockford, IL, United States) and 500 μL structure buffer was incubated in a water bath at 95°C for 2 min, followed by ice bath for 3 min. The magnetic beads (Dynabeads MyOne^TM^ streptavidin C1, Thermo Fisher Scientific) were resuspended with 50 μL bead suspension and incubated overnight at 4°C, followed by centrifugation at 3000 rpm for 3 min. After discarding the supernatant, the precipitate was rinsed three times with 500 μL wash buffer and incubated with 10 μL cell lysate at room temperature for 1 h. The incubated magnetic bead-RNA-protein mixture was centrifuged at a low speed after which the supernatant was collected and washed 3 times with 500 μL washing buffer. An additional 10 μL of cell lysate served as input. The expression of lncRNA AGAP2-AS1 was measured using RT-qPCR. Western blot analysis was conducted to detect Ago2 (1:500, ab186733, Abcam) expression.

### Construction of Stable Cell Lines

VCaP cells in each group were seeded into 6-well plates at a density of 10^5^ cells/well and treated with polyethylenimine (PEI) (Solarbio, Beijing, China) when cell confluence reached 80%. A total of 10 μg fluorescence-labeled lentiviral vector (pCDH; Miaolingbio. Inc., Hubei, China) expressing pCDNA-AGAP2-AS1, pCDNA-PDLIM5, miR-195-5p mimic, si-PDLIM5, si-AGAP2-AS1, or miR-195-5p inhibitor was well mixed with helper plasmids 7.5 μg PAX, and 5 μg pMD2G for 5 min. The mixture was diluted with 750 μL Opti-minimum essential medium (Opti-MEM) (Gibco Inc., Carlsbad, CA, United States). A total of 112.5 μg PEI was diluted with 750 μL Opti-MEM, mixed by means of gentle tapping, and then stood at room temperature for 5 min. The dilutions were then mixed in an even manner, left to stand for 20 min, and added to a 10 cm cell culture dish pretreated with 5 mL Dulbecco’s modified Eagle’s medium (DMEM) for 30 min, followed by culture at 37°C in a 5% CO_2_ incubator. After 6 h, the original medium was replaced by 8 mL of complete medium after which the cells were further cultured for 48 h, with the supernatant collected. After that, additional 8 mL complete medium was added to the cells, and 24 h later, the cell supernatant was collected.

The collected cell supernatant was centrifuged for 5 min and filtered through a 0.45 μm filter. Next, 30 mL filtrate was mixed with 7.5 mL of 5 × PEG 8000 mother liquor (8.776 g NaCl and 50 g PEG8000 dissolved in 200 mL ultrapure water, sterilized at 121°C for 30 min, stored at 4°C for usage) for 3–5 times (30 min/time), and stood overnight at 4°C. After the cells were centrifuged at 4000 g and 4°C for 40 min, the pellet was resuspended in 1 mL DMEM and stored at 4°C.

A total of 10^5^ cells were transduced with lentiviral vectors at a fixed titer and cultured for 24 h. Fluorescence intensity was then observed under a fluorescence microscope. Monoclonal cells were selected and amplified. The cells stably transduced with lentiviral vectors were collected accordingly.

### Xenograft Tumors in Nude Mice

The cells stably transduced with lentiviral vectors expressing pCDNA-AGAP2-AS1, PDLIM5 pCDNA-PDLIM5, miR-195-5p mimic, si-PDLIM5, si-AGAP2-AS1 or miR-195-5p inhibitor or respective NCs were resuspended with normal saline (5 × 10^7^ cells/mL) for subsequent use. A total of 60 specific pathogen-free (SPF) male nude mice (aged 7 weeks; weighing 20–22 g; Shanghai SLAC Laboratory Animal Co., Ltd., Shanghai, China) were selected for the purposes of this study. The nude mice were anesthetized with sodium pentobarbital, sterilized routinely, and administered with a subcutaneous injection with 200 μL cell suspension into the ventromedial area. After transplantation, the mice were raised under SPF conditions. The longest (A) and shortest (B) diameters of tumors were measured weekly. At the fourth week, the mice were euthanatized with the tumor mass subsequently determined. The volume (V) of the implanted tumors was calculated as: V = A^2^ × B/2 (mm^3^).

### Statistical Analysis

The data were analyzed using Statistical Product and Service Solutions (SPSS) 21.0 statistical software (IBM Corp., Armonk, NY, United States). Measurement data were expressed as the mean ± standard deviation of three independent experiments. Comparisons of data between two groups were conducted using *t*-test while data obeying normal distribution and homogeneity of variance were evaluated using an unpaired *t*-test. The data among multiple groups were tested using one-way analysis of variance (ANOVA), followed by Tukey’s test. Cell viability at different time points was compared using two-way ANOVA, while tumor volume data at different time points were compared using repeated measures ANOVA. A value of *p* < 0.05 was considered to be indicative of statistical significance.

## Results

### LncRNA AGAP2-AS1 Is Expressed Highly in Prostate Cancer Tissues

**FIGURE 1:**
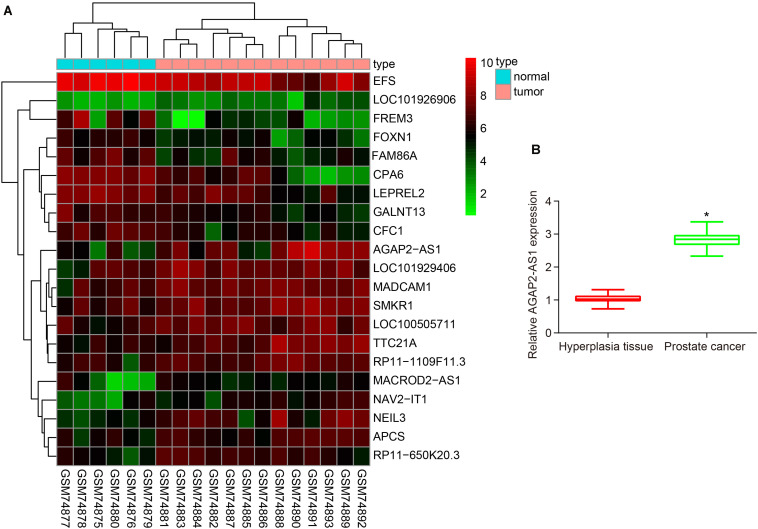
LncRNA AP2-AS1 is expressed at high levels in prostate cancer tissues. **(A)** LncRNA AGAP2-AS1 expression in the microarray GSE3325. **(B)** LncRNA AGAP2-AS1 expression examined by RT-qPCR in prostate cancer tissues (*n* = 50) and BPH tissues (*n* = 20). *p < 0.05 vs. the BPH tissues.

Analysis of prostate cancer-related microarray data GSE3325 revealed that lncRNA AGAP2-AS1 was a markedly up-regulated lncRNA in prostate cancer (Figure 1A). Additionally, the RT-qPCR results further illustrated an increase in the expression of lncRNA AGAP2-AS1 in prostate cancer tissues compared with BPH tissues (*p* < 0.05) (Figure 1B). Altogether, the results obtained provided evidence indicating that lncRNA AGAP2-AS1 was upregulated in prostate cancer tissues.

### Down-Regulation of lncRNA AGAP2-AS1 Inhibits Prostate Cancer Cell Proliferation, Migration, and Invasion *in vitro*

**FIGURE 2:**
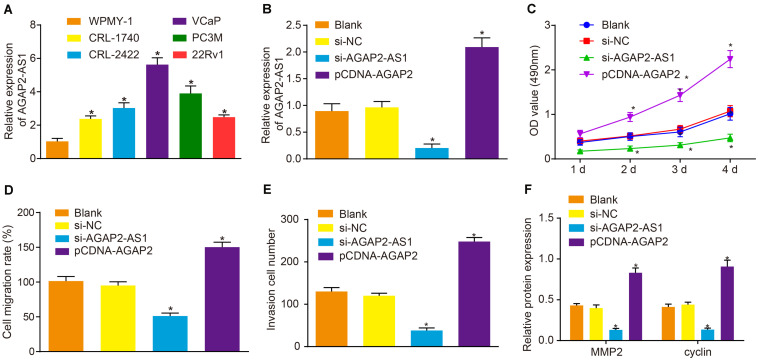
Down-regulation of lncRNA AGAP2-AS1 weakens proliferation, migration and invasion abilities of prostate cancer cells *in vitro*. **(A)** Expression of lncRNA AGAP2-AS1 examined by RT-qPCR in prostate cancer cell lines VCaP, 22Rv1, CRL-1740, CRL-2422, and PC3M, relative to that in normal human prostate cells WPMY-1. **(B)** LncRNA AGAP2-AS1 expression examined by RT-qPCR in VCaP cells transfected with si-AGAP2-AS1 or pCDH-AGAP2-AS1. **(C)** Viability of VCaP cells transfected with si-AGAP2-AS1 or pCDH-AGAP2-AS1 detected by CCK-8 assay. **(D)** Cell migration rate of cells transfected with si-AGAP2-AS1 or pCDH-AGAP2-AS1. **(E)** Quantitation of invasion ability of VCaP cells transfected with si-AGAP2-AS1 or pCDH-AGAP2-AS1. **(F)** Quantitation of cyclin and MMP2 proteins in VCaP cells transfected with si-AGAP2-AS1 or pCDH-AGAP2-AS1. In panel A, *p < 0.05 vs. normal human prostate cells WPMY-1. In panel C-I, *p < 0.05 vs. VCaP cells transfected with si-NC. The above results were all measurement data, expressed as mean ± standard deviation. One-way ANOVA was used for comparisons among multiple groups. Cell proliferation at different time points was analyzed by two-way ANOVA. The experiment was repeated 3 times.

RT-qPCR was initially employed to determine the expression of lncRNA AGAP2-AS1 in prostate cancer cell lines VCaP, 22Rv1, CRL-1740, CRL-2422, and PC3M, and normal human prostate cells WPMY-1. The results demonstrated that the expression of lncRNA AGAP2-AS1 was significantly higher in five prostate cancer cell lines than in WPMY-1, with the highest expression detected in VCaP cells (*p* < 0.05) (Figure 2A) which were therefore selected as the subject of following experiments. Next, to ascertain whether lncRNA AGAP2-AS1 regulates the biological functions of prostate cancer cells, the expression of lncRNA AGAP2-AS1 was down-regulated or up-regulated in VCaP cells. RT-qPCR results also displayed that lncRNA AGAP2-AS1 expression was markedly diminished by si-AGAP2-AS1 transfection, while elevations after pCDH-AGAP2-AS1 treatment were detected (*p* < 0.05) (Figure 2B). Cell viability was determined via CCK-8 assay (Figure 2C), cell migration using scratch test (Figure 2D) and cell invasion using Transwell assay (Figure 2E). The results displayed no notable difference between the cells transfected with empty plasmid and those transfected with si-NC (*p* > 0.05). Silencing of lncRNA AGAP2-AS1 significantly reduced cell viability, migration and invasion (*p* < 0.05), while overexpression of lncRNA AGAP2-AS1 led to an increase in cell viability, migration and invasion (*p* < 0.05). The expression of proliferation-related protein cyclin and migration-related protein MMP2 after lncRNA AGAP2-AS1 silencing was determined by means of western blot analysis, the result of which displayed no marked difference in the expression of those two proteins between the cells transfected with empty plasmid or si-NC (*p* > 0.05). The expression of cyclin and MMP2 was markedly down-regulated by transfection of si-AGAP2-AS1 (*p* < 0.05) but was largely up-regulated following transfection of pCDH-AGAP2-AS1 (*p* < 0.05) (Figure 2F). The aforementioned results support the conclusion that the progression of prostate cancer *in vitro* was restrained by lncRNA AGAP2-AS1 silencing.

### LncRNA AGAP2-AS1 Binds to miR-195-5p *in vitro*

**FIGURE 3:**
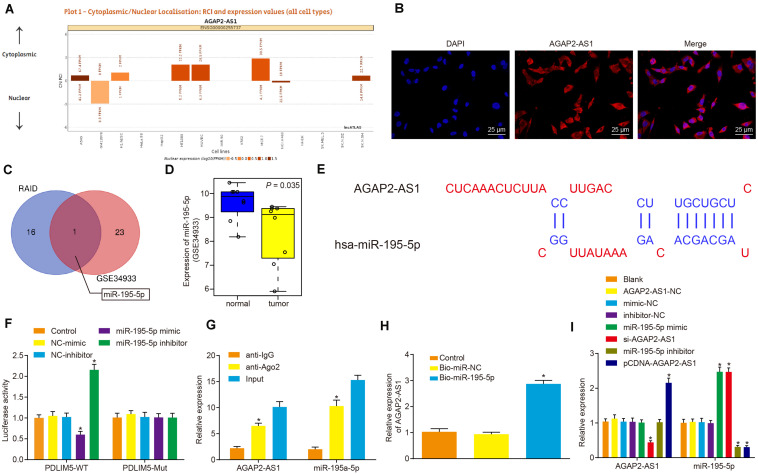
LncRNA AGAP2-AS1 binds to miR-195-5p and inhibits its expression *in vitro*. **(A)** Subcellular localization of lncRNA AGAP2-AS1 predicted by the lncATLAS website (http://lncatlas.crg.eu/). **(B)** Subcellular localization of AGAP2-AS1 in cells analyzed by FISH assay (×400). **(C)** Intersection of miRNAs possibly regulated by lncRNA AGAP2-AS1 predicted by the RAID database (http://www.rna-society.org/raid/search.html) and lowly-expressed miRNAs in prostate cancer in microarray data GSE3325. **(D)** miR-195-5p expression in prostate cancer in microarray data GSE3325. **(E)** The binding sites between AGAP2-AS1 and miR-195-5p predicted by the lncBase website. **(F)** LncRNA AGAP2-AS1 binding to miR-195-5p verified by dual-luciferase reporter assay in cells. The control group was used as a control (*p < 0.05 vs. the control group). **(G)** Binding of lncRNA AGAP2-AS1 to miR-195-5p analyzed by RIP assay in cells. The anti-IgG was used as a control (*p < 0.05 vs. anti-IgG). **(H)** Enrichment of lncRNA AGAP2-AS1 probed by miR-195-5p detected using RNA pull-down assay in cells. The control group was used as a control (*p < 0.05 vs. the control group). **(I)** miR-195-5p and lncRNA AGAP2-AS1 expression determined using RT-qPCR in VCaP cells after transfection with miR-195-5p mimic or inhibitor, pCDNA-AGAP2-AS1 or si-AGAP2-AS1. The blank group was used as a control. *p < 0.05 vs. VCaP cells without transfection. The above results were all measurement data expressed as mean ± standard deviation. One-way ANOVA was used for comparisons among multiple groups. The experiment was repeated 3 times.

LncRNA AGAP2-AS1 was predicted to be primarily located in the cytoplasm as per the lncATLAS website^3^ (Figure 3A), which was further verified following the analysis of VCaP cells under a fluorescence microscope by FISH assay (Figure 3B). Then, with the objective of identifying miRNAs that could be regulated by lncRNA AGAP2-AS1, the lncRNA AGAP2-AS1-binding miRNAs retrieved from RAID database^4^ were intersected with lowly-expressed miRNAs in prostate cancer microarray data GSE34933, which revealed miR-195-5p as a candidate miR (Figure 3C). The expression of miR-195-5p in prostate cancer and normal tissues from microarray data GSE34933 is depicted in Figure 3D. The lncBase website predicted the presence of the binding sites between lncRNA AGAP2-AS1 and miR-195-5p (Figure 3E). Meanwhile, the dual-luciferase reporter assay results demonstrated that the luciferase activity of the pMIR/AGAP2-AS1-WT system was considerably diminished in the miR-195-5p mimic group, while it was significantly increased in the miR-195-5p-inhibitor group (*p* < 0.05). The luciferase activity of the pMIR/AGAP2-AS1-Mut system was not affected in regard to AGAP2-AS1-binding miR-195-5p site (*p* > 0.05) (Figure 3F). RIP assay results illustrated that compared with input treatment the expression of miR-195-5p was dramatically reduced after lncRNA AGAP2-AS1 overexpression, while compared with IgG treatment, expression of miR-195a-5p was elevated (*p* < 0.05) (Figure 3G). RNA pull-down assay demonstrated that in contrast to the miR-NC group, the expression of Ago2 and lncRNA AGAP2-AS1 was significantly detected in the labeled miR-195a-5p group, which suggested lncRNA AGAP2-AS1 enrichment in samples pulled down by the miR-195a-5p probe (Figure 3H). RT-qPCR was performed to detect the expression of lncRNA AGAP2-AS1 and miR-195-5p after the cells were transfected with miR-195-5p mimic or inhibitor, pCDNA-AGAP2-AS1 or si-AGAP2-AS1. As illustrated in Figure 3I, with no significant difference detected in the expression of lncRNA AGAP2-AS1 and miR-195-5p among the cells transfected with empty plasmid, AGAP2-AS1-NC, mimic-NC and inhibitor-NC alone (*p* > 0.05). The expression of miR-195-5p was notably up-regulated in the cells transfected with miR-195-5p mimic or si-AGAP2-AS1 (*p* < 0.05), while dramatically down-regulated in the cells transfected with miR-195-5p inhibitor or pCDNA-AGAP2-AS1 (*p* < 0.05). The expression of lncRNA AGAP2-AS1 was considerably enhanced following transfection with pCDNA-AGAP2-AS1 (*p* < 0.05), but markedly reduced by transfection with si-AGAP2-AS1 (*p* < 0.05). In contrast, lncRNA AGAP2-AS1 expression was not influenced following transfection with either miR-195-5p mimic or miR-195-5p inhibitor (*p* < 0.05). Taken together, lncRNA AGAP2-AS1 could bind to miR-195-5p, thereby downregulating miR-195-5p expression *in vitro*.

### miR-195-5p Inhibits Prostate Cancer Cell Proliferation, Migration, and Invasion by Targeting PDLIM5 *in vitro*

**FIGURE 4:**
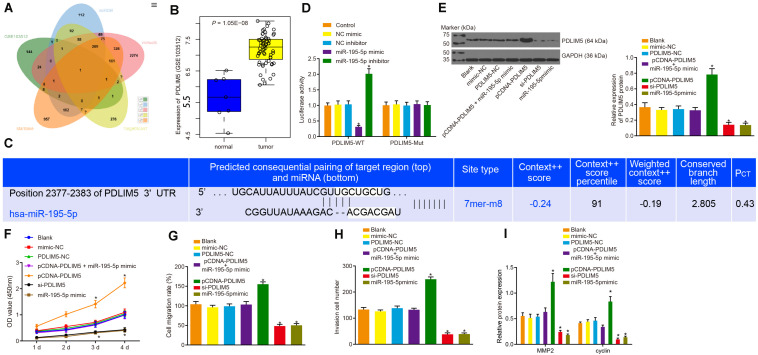
miR-195-5p restrains the PDLIM5 expression, thereby arresting the proliferation, migration and invasion abilities of prostate cancer cells *in vitro*. **(A)** Intersection of predicted target genes of miR-195-5p by miRDB (http://www.mirdb.org/), TargetScan7 (http://www.targetscan.org/vert_71/), starBase (http://starbase.sysu.edu.cn/index.php) and mirwalk (http://mirwalk.umm.uni-heidelberg.de/) databases and highly-expressed genes in prostate cancer in microarray data GSE3325. **(B)** PDLIM5 expression in prostate cancer in microarray data GSE3325. **(C)** The binding sites between miR-195-5p and PDLIM5 predicted by the Targetscan website. **(D)** miR-195-5p binding to PDLIM5 verified using luciferase reporter assay in cells. **(E)** Representative Western blots of PDLIM5 protein and its quantitation in VCaP cells transfected with pCDNA-PDLIM5, si-PDLIM5, miR-195-5p mimic or pCDNA-PDLIM5 + miR-195-5p mimic. **(F)** Cell viability detected by CCK-8 assay upon transfection with pCDNA-PDLIM5, si-PDLIM5, miR-195-5p mimic or pCDNA-PDLIM5 + miR-195-5p mimic. **(G)** Quantitation of cell migration ability upon transfection with pCDNA-PDLIM5, si-PDLIM5, miR-195-5p mimic or pCDNA-PDLIM5 + miR-195-5p mimic. **(H)** Cell invasion ability upon transfection with pCDNA-PDLIM5, si-PDLIM5, miR-195-5p mimic or pCDNA-PDLIM5 + miR-195-5p mimic. **(I)** MMP2 and cyclin proteins in VCaP cells transfected with pCDNA-PDLIM5, si-PDLIM5, miR-195-5p mimic or pCDNA-PDLIM5 + miR-195-5p mimic. *p < 0.05 vs. VCaP cells without transfection. The above results were all measurement data, expressed as mean ± SD. One-way ANOVA was used for comparisons among multiple groups. Cell proliferation at different time points was analyzed by two-way ANOVA. The experiment was repeated 3 times.

The target genes of miR-195-5p predicted by the miRDB^5^, TargetScan7^6^, starBase^7^, and mirwalk^8^ databases were intersected with the highly expressed genes in prostate cancer from microarray data GSE103512, the result of which suggested that PDLIM5 was a target gene of miR-195-5p (Figure 4A). The expression of PDLIM5 in prostate cancer and normal tissues from microarray data GSE103512 are depicted in Figure 4B. The binding sites of miR-195-5p in the 3’UTR of PDLIM5 mRNA predicted by the Targetscan website (Figure 4C). Additionally, the dual luciferase reporter assay revealed that the luciferase activity of the pMIR/PDLIM5-WT system was dramatically decreased in the miR-195-5p mimic group, but it was increased in the miR-195-5p-inhibitor group (*p* < 0.05); with no significant difference in terms of luciferase activity of the mutated pMIR/PDLIM5-Mut system at the PDLIM5-binding miR-195-5p site (*p* > 0.05) identified (Figure 4D). Next, VCaP cells were transfected with pCDNA-PDLIM5, si-PDLIM5, or miR-195-5p mimic to identify the regulatory effect of miR-195-5p on PDLIM5. Western blot analysis revealed that the protein expression of PDLIM5 was not affected by transfection with PDLIM5-NC, mimic-NC or co-transfection with pCDNA-PDLIM5 and miR-195-5p mimic (*p* > 0.05). The expression of PDLIM5 was markedly up-regulated by transfection with pCDNA-PDLIM5, while notably down-regulated following transfection with si-PDLIM5 or miR-195-5p mimic (*p* < 0.05) (Figure 4E), indicating that miR-195-5p could target PDLIM5 and negatively regulate its expression in VCaP cells.

The regulatory effects on the functions of VCaP cells were subsequently evaluated. The results of CCK-8 assay (Figure 4F), scratch test (Figure 4G) and Transwell assay (Figure 4H) provided evidence demonstrating that cell viability, migration and invasion abilities were apparently increased by transfection of pCDNA-PDLIM5, but was dramatically decreased by transfection of si-PDLIM5 or miR-195-5p mimic (*p* < 0.05). Also, western blot analysis results indicated there was an increase in the protein expression of MMP2 and cyclin resulted from PDLIM5 overexpression, and a reduction caused by PDLIM5 silencing or miR-195-5p enhancement (Figure 4I). In summary, miR-195-5p could target PDLIM5 and suppress the proliferation, migration and invasion abilities of prostate cancer cells *in vitro*.

### Knockdown of lncRNA AGAP2-AS1 Inhibits PDLIM5 and Impedes Prostate Cancer Cell Proliferation, Migration, and Invasion Through Upregulation of miR-195-5p *in vitro*

**FIGURE 5:**
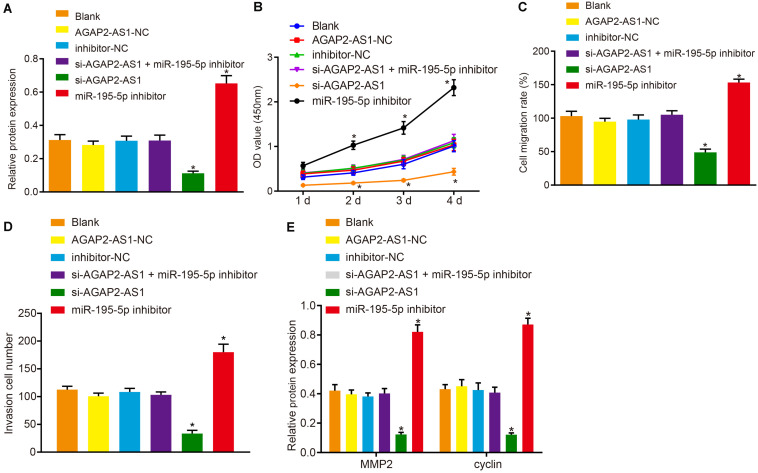
Knockdown of lncRNA AGAP2-AS1 elevates miR-195-5p expression and down-regulates PDLIM5 expression, thereby prohibiting the proliferation, migration and invasion abilities of prostate cancer cells *in vitro*. VCaP cells were transfected with si-AGAP2-AS1 and miR-195-5p inhibitor either alone or in combination. **(A)** PDLIM5 protein expression in VCaP cells determined by western blot analysis. **(B)** Cell viability assessed by CCK-8 assay. **(C)** Cell migration ability detected by Scratch test. **(D)** Cell invasion ability measured by Transwell assay. **(E)** MMP2 and cyclin protein expression in VCaP cells determined by western blot analysis. *p < 0.05 vs. VCaP cells without transfection. The above results were all measurement data, expressed as mean ± SD. One-way ANOVA was used for comparisons among multiple groups. Cell viability at different time points was analyzed by two-way ANOVA. The experiment was repeated 3 times.

Next, to elucidate the regulatory mechanism of lncRNA AGAP2-AS1 in the progression of prostate cancer, the cells were transfected with si-AGAP2-AS1 and miR-195-5p inhibitor either alone or in combination. Western blot results revealed there to be no significant difference in terms of PDLIM5 protein expression between the cells transfected with AGAP2-AS1-NC, inhibitor-NC, both si-AGAP2-AS1 and miR-195-5p inhibitor, and the cells without transfection (*p* > 0.05). Besides, PDLIM5 expression was dramatically decreased in cells transfected with si-AGAP2-AS1, while marked elevations were detected in the cells transfected with miR-195-5p inhibitor (*p* < 0.05, Figure 5A). Furthermore, CCK-8 assay (Figure 5B), scratch test (Figure 5C), Transwell assay (Figure 5D) and western blot data (Figure 5E) suggested that cell viability, migration and invasion abilities as well as the expression of MMP2 and cyclin proteins were evidently enhanced following transfection of miR-195-5p inhibitor, while it was largely diminished following transfection of si-AGAP2-AS1 (*p* < 0.05). Collectively, lncRNA AGAP2-AS1 silencing inhibited the expression of PDLIM5, and weakened the proliferation, migration and invasion of prostate cancer cells *via* miR-195-5p up-regulation *in vitro*.

### LncRNA AGAP2-AS1 Knockdown Suppresses Tumor Growth Through Downregulation of PDLIM5 and Up-Regulation of miR-195-5p *in vivo*

**FIGURE 6:**
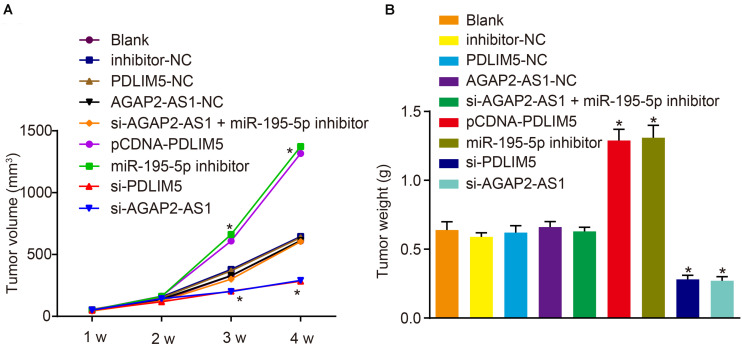
Reduction of lncRNA AGAP2-AS1 expression prohibits tumor growth in nude mice by enhancing miR-195-5p and down-regulating PDLIM5. Mice were injected with VCaP cells with transfection or stably transfected with pCDNA-PDLIM5, miR-195-5p inhibitor, si-PDLIM5, si-AGAP2-AS1, both si-AGAP2-AS1, and miR-195-5p inhibitor or their corresponding controls. **(A)** Volume of implanted tumors in nude mice every 1 week. **(B)** Weight of implanted tumors in nude mice. *p < 0.05 vs. mice injected with non-transfected VCaP cells. The above results were all measurement data, expressed as mean ± standard deviation. One-way ANOVA was used for comparisons among multiple groups. Tumor volume at different time points was analyzed by repeated measures ANOVA, n = 6 for mice upon each treatment.

Xenograft nude mouse models were established in order to elucidate the role of lncRNA AGAP2-AS1 in prostate cancer *in vivo*. The results revealed that the tumor volume and weight were considerably elevated in the mice injected with cells stably transfected with pCDNA-PDLIM5 or miR-195-5p inhibitor in the 4th week, which were markedly diminished following PDLIM5 silencing or lncRNA AGAP2-AS1 silencing (*p* < 0.05) (Figures 6A,B). This indicated that tumor growth was suppressed by lncRNA AGAP2-AS1 silencing but promoted by inhibition of miR-195-5p. Taken together, the aforementioned findings demonstrated that silencing of lncRNA AGAP2-AS1 could enhance miR-195-5p expression, but down-regulate PDLIM5 expression, thereby deterring implanted tumor growth *in vivo*.

## Discussion

Over the past two decades, prostate cancer has witnessed a progressive increase in its global incidence, with studies highlighting factors such as a progressive aging population, the improvement in diagnostic techniques and a higher intensity screening of prostate cancer as key contributors (Plata Bello and Concepcion Masip, 2014; Chen et al., 2016). Owing to the limited knowledge regarding the function of lncRNAs in the metastatic prostate cancer, it’s important to clarify the genetic abnormalities and underlying molecular mechanisms associated with the progression of prostate cancer (Quigley et al., 2018). During the present study, our results demonstrated that lncRNA AGAP2-AS1 silencing could inhibit PDLIM5 expression, and impede prostate cancer development and progression through the up-regulation of miR-195-5p expression.

A key initial finding of our study illustrated that lncRNA AGAP2-AS1 expression was elevated in prostate cancer tissues relative to BPH tissues. Moreover, lncRNA AGAP2-AS1 expression was markedly up-regulated in the prostate cancer cell lines VCaP, 22Rv1, CRL-1740, CRL-2422, and PC3M. Several aberrantly expressed prostate-specific lncRNAs, including prostate cancer antigen 3 (PCA3), prostate cancer gene expression marker 1 (PCGEM1), and prostate cancer associated ncRNA transcript 1 (PCAT1) have been implicated in prostate carcinogenesis (Walsh et al., 2014). Existing literature has indicated that the expression of lncRNA AGAP2-AS1 is up-regulated in breast cancer; high lncRNA AGAP2-AS1 expression has been shown to expedite cancer cell growth and inhibits apoptosis (Dong et al., 2018). Similarly, lncRNA AGAP2-AS1 was overexpressed in gastric cancer cells and tissues, while lncRNA AGAP2-AS1 silencing markedly restrained the biological abilities of gastric cancer cells and tumor growth *in vivo* (Qi et al., 2017). Additionally, a previous report suggested that the expression of lncRNA AGAP2-AS1 was increased in NSCLC, and that the knockdown of lncRNA AGAP2-AS1 prevented proliferation, invasion and migration of cells (Li et al., 2016). The aforementioned findings are largely consistent with our findings whereby the repression of lncRNA AGAP2-AS1 was found to inhibit the proliferation, migration and invasion abilities of prostate cancer cells. Moreover, the expression of proliferation-related protein cyclin and migration-related protein MMP2 was evidently down-regulated by silencing lncRNA AGAP2-AS1, which was largely indicative of restrained prostate cancer cell proliferation.

Furthermore, this study found that lncRNA AGAP2-AS1 could bind to miR-195-5p and act as a suppressor of miR-195-5p inhibiting its expression. LncRNAs have been reported to regulate the prostate cancer progression through sponging miRNAs (Xu et al., 2018). A circRNA circAGFG1 sponges miR-195-5p to accelerate the development of triple-negative breast cancer (Yang et al., 2019). LncRNA XIST also motivates osteosarcoma progression *via* sponging miR-195-5p (Yang et al., 2018). Consistent with the findings of the current study, lncRNA AGAP2-AS1 has been previously reported to sponge miR-195-5p leading to a reduction in the expression of miR-195-5p in esophageal cancer cells (Shen et al., 2020). Meanwhile, our results illustrated that miR-195-5p was poorly expressed in prostate cancer and that its upregulation could repress prostate cancer cell proliferation, migration and invasion. Consistently, previous research has also indicated that prostate cancer cells exhibit poor expression of miR-195-5p, and that its upregulation limits cancer cell proliferation (Xing et al., 2020). The overexpression of miR-195 has been reported to markedly suppress prostate cancer cell proliferation and tube formation by downregulating proline-rich protein 11 (PRR11) expression (Cai et al., 2018). Furthermore, miR-195-5p has been suggested to function as a tumor suppressor in prostate cancer development and progression as its overexpression represses the migration and invasion of prostate cancer cells by regulating Fra-1 (Wu et al., 2015). Based on these findings, lncRNA AGAP2-AS1 silencing could potentially impede the progression of prostate cancer through up-regulation of miR-195-5p.

The current study further demonstrated that lncRNA AGAP2-AS1 knockdown triggered an increase in the expression of miR-195-5p to suppress the expression of PDLIM5. The potential of PDLIM5 as an oncogene has been implicated in the progression of prostate cancer where its silencing leads to restrained prostate cancer colony formation and induced apoptosis, as well as confirmed inhibition of tumor growth in nude mice (Liu et al., 2017). A previous study revealed that siRNA-mediated PDLIM5 silencing inhibited gastric cancer cell proliferation and colony formation, and enhanced cell apoptosis (Li et al., 2015). Another literature continues to indicate that miRNAs play an important role in regulating cancer cell growth, invasion and metastasis by inhibiting the expression of their targets (Chan and Wang, 2015). miR-195 has been reported to act as a suppressor of prostate cancer by targeting Fra-1 (Wu et al., 2015). Similarly, our study also suggested that miR-195-5p inhibited prostate cancer progression by inhibiting its target PDLIM5. Most importantly, it was confirmed that down-regulation of lncRNA AGAP2-AS1 increased miR-195-5p expression and reduced PDLIM5 expression, thus inhibiting the growth of implanted tumors *in vivo*.

## Conclusion

**FIGURE 7:**
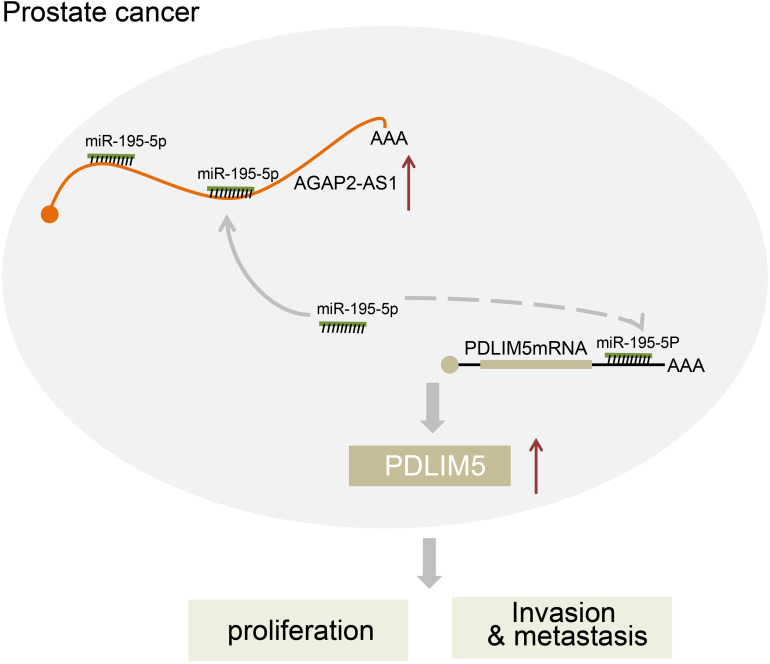
The regulatory network of lncRNA AGAP2-AS1/miR-195-5p/PDLINM5 involved in the progression of prostate cancer. LncRNA AGAP2-AS1 increases the expression of PDLINM5 by binding to miR-195-5p and inducing the resultant inhibition, thus stimulating the proliferation, migration and invasion of prostate cancer cells.

In conclusion, the key findings of our study presented evidence suggesting that the down-regulation of lncRNA AGAP2-AS1 inhibited PDLIM5 expression, which ultimately impedes prostate cancer development and progression through up-regulating miR-195-5p expression (Figure 7). Therefore, our study highlights lncRNA AGAP2-AS1 as a novel biomarker for prostate cancer prognosis and as a promising therapeutic target. Nonetheless, further studies with more other experimental subjects or methods are desired before lncRNAs such as lncRNA AGAP2-AS1 can be used in clinical applications.

## Data Availability Statement

All datasets generated for this study are included in the article/supplementary material, further inquiries can be directed to the corresponding author/s.

## Ethics Statement

The study protocol was approved by the Ethics Committee of Qujing Affiliated Hospital of Kunming Medical University. All patients or their family were informed of the research purposes and provided their written informed consent. The experiment procedures accorded with the Declaration of Helsinki. The animal experiments were conducted with the approval of the Animal Ethics Committee of Qujing Affiliated Hospital of Kunming Medical University.

## Author Contributions

FC and PX designed the study. ML, SW, and WW collated the data, carried out data analyses and produced the initial draft of the manuscript. HZ, TS and CX contributed to drafting the manuscript. All authors have read and approved the final submitted manuscript.

## Conflict of Interest

The authors declare that the research was conducted in the absence of any commercial or financial relationships that could be construed as a potential conflict of interest.

## Footnotes

1 http://www.ncbi.nlm.nih.gov/geo

2 http://lncatlas.crg.eu/

3 http://www.ncbi.nlm.nih.gov/gene

4 http://www.rna-society.org/raid/search.html

5 http://www.mirdb.org/

6 http://www.targetscan.org/vert_71/

7 http://starbase.sysu.edu.cn/index.php

8 http://mirwalk.umm.uni-heidelberg.de/

## Abbreviations

3 ′ UTR: 3 ′ untranslated region
AGAP2-AS1: AGAP2 antisense RNA 1
Ago2: Argonaute 2
ANOVA: analysis of variance
BCA: bicinchoninic acid
BPH: benign prostatic hyperplasia
BSA: bovine serum albumin
CCK-8: cell counting kit-8
cDNA: complementary DNA
ceRNAs: competing endogenous RNAs
DAB: diaminobenzidine
DMEM: Dulbecco’s modified Eagle’s medium
DTT: dithiothreitol
ECL: enhanced chemiluminescence
EDTA: ethylene diamine tetraacetic acid
FBS: fetal bovine serum
GEO: Gene Expression Omnibus
IgG: immunoglobulin G
ISUP: International Society of Urological Pathology
lncRNAs: long non-coding RNAs
miR-195-5p: microRNA-195-5p
miRNAs/miRs: microRNAs
MMP2: matrix metalloproteinase 2
mut: mutant
NC: negative control
NSCLC: non-small cell lung cancer
OD: optical density
Opti-MEM: Opti-minimum essential medium
PBS: phosphate buffered saline
PCA3: prostate cancer antigen 3
PCAT1: prostate cancer associated ncRNA transcript 1
PCGEM1: prostate cancer gene expression marker 1
PEI: polyethylenimine
PRR11: proline-rich protein 11
RAID: redundant arrays of inexpensive disks
RIP: RNA-binding protein immunoprecipitation
RIPA: radioimmunoprecipitation assay
RPMI: Roswell Park Memorial Institute
RT-qPCR: Reverse transcription quantitative polymerase chain reaction
siRNA: small interfering RNA
SP: streptavidin-perosidase
SPF: specific pathogen-free
SPSS: Statistical Product and Service Solutions
wt: wild type.
